# A Multiplex Real‐Time PCR for Qualitative Detection of Mutton, Pork, Chicken, and Duck in Processed Meat Products

**DOI:** 10.1002/fsn3.70590

**Published:** 2025-07-14

**Authors:** Lijuan Chang, Chengping Fu, Mengxue Deng, Yuanhong Li

**Affiliations:** ^1^ Institute of Remote Sensing and Digital Agriculture Sichuan Academy of Agricultural Sciences Chengdu China

**Keywords:** meat adulteration, multiplex real‐time PCR, processed meat products, qualitative detection

## Abstract

Economically motivated adulteration (EMA) is a persistent global issue in the meat industry. This study aimed to develop a qualitative detection method for mutton, pork, chicken, and duck meat based on nuclear genes. The specificity of the method was validated by amplifying DNA from 23 samples including animal, plant, and microbial mixtures. Accuracy was confirmed by testing 11 reference samples, which produced the expected results. Sensitivity tests demonstrated a limit of detection as low as 10 copies for mutton, pork, and chicken, and 20 copies for duck. To assess the applicability of this method to real‐world samples, 35 processed meat products were tested using the established multiplex real‐time polymerase chain reaction (PCR) method. Labeling information for 34 of the 35 products matched the detection results, with one exception: a mutton roll contained no detectable mutton. Therefore, the developed multiplex real‐time PCR method demonstrated good specificity, accuracy, and sensitivity, making it a reliable tool for the qualitative detection of mutton, pork, chicken, and duck in processed meat products.

## Graphical abstract

This study aimed to develop a multiplex real‐time PCR method for the qualitative detection of pork, chicken, and duck adulteration in mutton products. The method exhibited high specificity, accuracy, and sensitivity through method validation. To evaluate its applicability, we analyzed 35 commercial processed meat products, and the results confirmed the assay's robustness and reliability for meat authenticity testing.

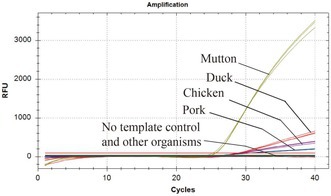

## 1 Introduction

In recent years, incidents of meat adulteration have become increasingly frequent worldwide. Notable examples include the “horse meat scandal” in Europe (Hsieh and Ofori 2014), the adulteration of beef meatballs with buffalo meat in Malaysia (Hossain et al. 2017), and the meat cartel scandal in Johor Baharu (Md Ariffin and Abdul Hamid 2021; Md Ariffin et al. 2022). Despite the establishment of laws and regulations mandating accurate labeling of meat products, adulteration persists, severely hindering the healthy development of the meat industry (Barnard and O'Connor 2017; Liu et al. 2021). Mutton production and consumption have steadily increased over the years due to its high protein content, low fat, and low cholesterol. However, its high price and popularity have led some producers to adulterate processed mutton products by adding cheaper meats such as pork, chicken, duck or even inedible meats to increase profits (Wang et al. 2022). Such adulteration negatively affects food safety, fair trade, and religious practices (Jiang et al. 2022). Therefore, developing a specific and accurate method to screen for undeclared meat components in mutton products is essential.

Detection methods for meat adulteration are mostly based on the analysis of the molecular structure of proteins, species‐specific DNA sequences, or fatty acid composition. These methods can be divided into: physical methods, such as Raman spectroscopy (Logan et al. 2021), near‐infrared spectroscopy (Leng et al. 2020), hyperspectral imaging (Masithoh et al. 2024) and laser‐induced breakdown spectroscopy (Sezer et al. 2022). Chemical methods mainly involve mass spectrometry (Nalazek‐Rudnicka et al. 2022) and chromatography‐based techniques, often coupled with other analytical tools (Zhang et al. 2022; Windarsih et al. 2024). Molecular biology methods, such as enzyme‐linked immunosorbent assay (Zvereva et al. 2015), loop‐mediated isothermal amplification (Sul et al. 2019), droplet digital PCR (Vishnuraj et al. 2021), sequencing (Cottenet et al. 2020) and real‐time PCR (Amaral et al. 2017; Burns and Nixon 2022). Among these, real‐time PCR is one of the most widely used techniques for meat testing. Compared with real‐time PCR, multiplex real‐time PCR is more time‐ and cost‐efficient because it can identify multiple targets in a single PCR reaction (Hossain et al. 2023; Li et al. 2022). Therefore, multiplex real‐time PCR is suitable for qualitative screening of meat components in processed meat products. Selecting the proper target genes is crucial for developing a reliable real‐time PCR method. Previous studies have used mitochondrial genes to qualitatively or quantitatively detect meat components (Kim and Kim 2019; Li et al. 2022, 2019; Wang et al. 2022). Several studies have shown that mitochondrial gene‐based methods are highly sensitive. However, the number of mitochondrial gene copies can vary, leading to unstable sensitivity (Floren et al. 2015). Therefore, recent efforts have focused on using nuclear genes to develop real‐time PCR methods, particularly for quantitative detection. Despite their potential, suitable nuclear genes are still limited (Iwobi et al. 2015, 2017; Li et al. 2021; Wang et al. 2019, 2021). To date, there have been no reports on using multiplex real‐time PCR with nuclear genes to screen for pork, chicken, or duck in processed mutton products.

In this study, we developed a multiplex real‐time PCR method to qualitatively detect mutton, pork, chicken, and duck meat in processed meat products. The primers and probes were designed based on species‐specific sequences of nuclear genes: *putative HERV‐K_5q13.3 provirus ancestral Env polyprotein‐like* (*PHPAP*), *prion protein* (*PRNP*), *transforming growth factor beta 3* (*TGF‐β3*), and *beta‐actin* (*ACTB*). We tested the specificity, accuracy, and sensitivity to ensure reliability of the multiplex real‐time PCR method established in this study. The detection results for 35 processed meat products indicated that this method is reliable for combating mutton adulteration in the meat industry.

## 2 Materials and Methods

### 2.1 Samples

Meat powder certified reference materials (CRMs) of sheep ( *Ovis aries* ), goat ( *Capra hircus* L), beef ( *Bos taurus* ), pork (*Sus scrofa domestica*), chicken ( *Gallus gallus* ), duck ( *Anas platyrhynchos* ), horse ( *Equus caballus* ), dog ( *Canis lupus familiaris* ), donkey ( *Equus asinus* ), and Sika deer ( *Cervus nippon* Temminck) were purchased from the National Institute of Metrology, China. Positive control materials of rabbit (*Leporidae*), cat ( *Felis catus* ), goose ( *Anser anser* ), deer (*Cervus axis*), marten ( *Neovison vison* ), pigeon ( *Columba livia* ), and quail ( *Coturnix coturnix* ) were purchased from the Chinese Academy of Inspection and Quarantine. Fresh muscle tissues of sheep ( *O. aries* ), goat (*C. hircus L*), pork ( *S. scrofa* ), chicken ( *G. gallus* ), duck ( *A. platyrhynchos* ), carp ( *Cyprinus carpio* ), prawn ( *Penaeus orientalis* ), rice field eel ( *Monopterus albus* ), and bullfrog ( *Rana catesbeiana* ) were purchased from local markets in Chengdu, Sichuan. Plant mixtures included rice ( *Oryza sativa* ), corn ( *Zea mays* ), soybean ( *Glycine max* ), rapeseed ( *Brassica napus* ), pepper ( *Capsicum annuum* ), and wheat ( *Triticum aestivum* ). The microbial mixture contained *Salmonella typhimurium* , *Escherichia coli* , *Staphylococcus aureus* , and *Bacillus cereus* .

The reference samples for the accuracy tests were prepared in the laboratory using the CRMs of mutton, pork, chicken, and duck, numbered from A1 to A11. The composition of the reference samples was as follows: A1 (mutton_0.1 g_/pork_0.1 g_/chicken_0.1 g_/duck_0.1 g_), A2 (mutton_0.2 g_/pork_0.2 g_/chicken_0.2 g_), A3 (mutton_0.2 g_/pork_0.2 g_/ duck_0.2 g_), A4 (mutton_0.2 g_/chicken_0.2 g_/duck_0.2 g_), A5 (pork_0.2 g_/chicken_0.2 g_/duck_0.2 g_), A6 (mutton_0.2 g_/pork _0.2 g_), A7 (mutton _0.2 g_/chicken _0.2 g_), A8 (mutton _0.2 g_/duck _0.2 g_), A9 (pork _0.2 g_/chicken_0.2 g_), A10 (pork_0.2 g_/duck_0.2 g_), and A11 (chicken_0.2 g_/ duck_0.2 g_). The reference samples for the sensitivity tests were prepared in the laboratory according to different genomic equivalent (GE) concentration gradients, numbered D1–D4. The composition of the reference samples was as follows: D1 (mutton 20 GE/μL, pork 20 GE/μL, chicken 20 GE/μL, duck 20 GE/μL, beef 20,000 GE/μL), D2 (mutton 10 GE/μL, pork 10 GE/μL, chicken 10 GE/μL, duck 10 GE/μL, beef 10,000 GE/μL), D3 (mutton 5 GE/μL, pork 5 GE/μL, chicken 5 GE/μL, duck 5 GE/μL, beef 5000 GE/μL), and D4 (mutton 1 GE/μL, pork 1 GE/μL, chicken 1 GE/μL, duck 1 GE/μL, beef 1000 GE/μL). To avoid cross‐contamination, the CRMs of each animal were weighed separately and then mixed in mass proportions as described above.

A total of 35 processed meat products, including mutton (*n* = 2), mutton rolls (*n* = 14), shish kebab (*n* = 3), ham (*n* = 2), luncheon meat (*n* = 4), bacon (*n* = 1), chicken sausage (*n* = 2), chicken breast (*n* = 2), chicken thigh (*n* = 1), hot dog meat (*n* = 1), duck neck (*n* = 1), duck wing (*n* = 1), and duck leg (*n* = 1), were purchased from local markets.

### 2.2 DNA Isolation

DNA was extracted according to the method described by Chang et al. (2024). The purity and concentration of DNA were measured using a NanoDrop ND‐1000 UV Spectrophotometer (Thermo Scientific, USA). The 260/280 nm ratio of DNA was between 1.8 and 2.0, and the 260/230 nm ratio was greater than or equal to 2.0. The DNA samples meeting these criteria were subsequently diluted to 50 ng / μL^−1^.

### 2.3 Screening of Target Genes

A multi‐stage screening strategy was employed to identify nuclear genes suitable for species‐specific detection. Initially, candidate single‐copy nuclear genes were retrieved through literature review and mining of publicly available genome databases. Multiple sequence alignments were then performed to identify regions exhibiting high interspecies variation and species‐specific sequences suitable for primer and probe design. Candidate genes lacking sufficient interspecies divergence or appropriate sites for oligonucleotide design were excluded. Only gene targets demonstrating high species specificity, good sequence conservation within species, and favorable amplification efficiency were retained for further assay development. As a result, the *PHPAP*, *PRNP*, *TGF‐β3*, and *ACTB* genes were ultimately selected as target genes for the specific detection of mutton, pork, chicken, and duck, respectively, in the multiplex assay.

### 2.4 Primers and Probes

Species‐specific sequences of target genes were identified using sequence alignment, and the primers and probes were designed based on these sequences using the Primer Express software version 3.0 (Applied Biosystems, Foster City, CA, USA). The specificity of the primers and probes was verified using Primer‐BLAST on the NCBI platform (https://blast.ncbi.nlm.nih.gov/). The probes for mutton, pork, chicken, and duck were labeled with fluorescence reporting groups (VIC, FAM, Cy5, and Texas) at the 5′ end, and fluorescence quenching groups (BHQ1, BHQ1, BHQ3, and BHQ2) at the 3′ end. All primers and probes were synthesized by Sangon Biotech Co. Ltd. (Shanghai, China). The details of the primers and probes used are shown in Table 1.

**TABLE 1:** Information of primers and probes used in this study.

Name	Target gene	Sequences (5′–3′)	Amplicon size (bp)	GeneBank ID
Ovi‐F Ovi‐R Ovi‐P	*PHPAP*	ATGCTTGCTGTGGTCATTGCT TGTAACGCAAGGCCTGCTACA VIC‐TTCTCGCAGTCGCATCA‐BHQ1	80 bp	XM_040104.1
Sus‐F Sus‐R Sus‐P	*PRNP*	GCAAGGCCAGGGATCGA AGCAGAAACCAGCCATGGATT FAM‐CCGCGTCCTCATGG‐BHQ1	59 bp	XM_005672669.2
Gal‐F Gal‐R Gal‐P	*TGF‐β3*	CGTCCCTCACTTCCATATTGATG ATCTTCACTGTCAATGCCTTCACA Cy5‐CCTCTTTGTGGATCCTGT‐BHQ3	71 bp	NC_052536.1
Ana‐F Ana‐R Ana‐P	*ACTB*	CAGTCCCCCCTGCCTAGGA TCAGTGTACAGGTAGCCCCTCTCT Texas‐AAGTGCCAGTATGCGGG‐BHQ2	63 bp	NC_051786.1

### 2.5 Multiplex Real‐Time PCR Conditions

Multiplex real‐time PCR amplification with TaqMan probes was performed using a CFX96 Touch (Bio‐Rad, Hercules, CA, USA). The primer and probe concentrations and annealing temperatures were optimized based on CT value. The reaction system contained 200 GE of DNA template. The concentration gradient of primers and probes included CG1: 0.04 μM (Ovi‐F/R, Sus‐F/R, Gal‐F/R), 0.02 μM (Ovi‐P, Sus‐P.Gal‐P), 0.08 μM (Ana‐F/R), 0.04 μM (Ana‐P); CG2: 0.12 μM (Ovi‐F/R, Sus‐F/R, Gal‐F/R), 0.06 μM (Ovi‐P, Sus‐P, Gal‐P), 0.16 μM (Ana‐F/R), 0.08 μM (Ana‐P); CG3: 0.20 μM (Ovi‐F/R, Sus‐F/R, Gal‐F/R), 0.10 μM (Ovi‐P, Sus‐P, Gal‐P), 0.24 μM (Ana‐F/R), 0.12 μM (Ana‐P); CG4: 0.28 μM (Ovi‐F/R, Sus‐F/R, Gal‐F/R), 0.14 μM (Ovi‐P, Sus‐P, Gal‐P), 0.32 μM (Ana‐F/R), 0.16 μM (Ana‐P). The temperature gradient included TG1: 56°C, TG2: 58°C, TG3: 60°C, TG4: 62°C. All samples were analyzed in triplicate and NTC served as the blank control. Fluorescence signals were analyzed, and cycle threshold (Ct) values were automatically calculated using the Bio‐Rad CFX Manager software version 3.1.

### 2.6 Specificity Test

To test the specificity of the established multiplex real‐time PCR method, genomic DNA from 21 animal species, plant mixtures, and microbial mixtures was used as a template for the multiplex real‐time PCR assay. The reaction system contained 50 ng of DNA template. All samples were analyzed in triplicate. Molecular‐grade water was used in place of the template in the no‐template control (NTC).

### 2.7 Accuracy Test

Eleven reference samples A1 to A11 were mixed with CRM of mutton, pork, chicken, and duck. The multiplex real‐time PCR was performed using 50 ng DNA as a template. All samples were analyzed in triplicate. Molecular‐grade water was used in place of the template in the NTC. The accuracy of the multiplex real‐time PCR assay was evaluated by comparing the results with the predicted meat components.

### 2.8 Sensitivity Test

To evaluate the sensitivity of the established multiplex real‐time PCR assay, DNA from mutton, pork, chicken, duck, and beef CRMs was mixed and diluted to create a series of DNA concentration gradient samples numbered D1 to D4. One microliter of the mixture was used as a template for the multiplex real‐time PCR. Each concentration point was repeated sixty times according to the European Network of GMO Laboratories (ENGL) criteria (ENGL 2015). Molecular‐grade water was used in the NTC.

### 2.9 Applicability Test

To test the applicability of the established multiplex real‐time PCR method for analyzing practical samples, 35 processed meat products containing mutton, pork, chicken, and duck were collected from the market. DNA was extracted and used as the template for multiplex real‐time PCR. All PCR reactions were repeated three times, with molecular‐grade water as the NTC. The detection results were compared with labeling information to validate the applicability of the multiplex real‐time PCR method.

## 3 Results

### 3.1 Development of Multiplex Real‐Time PCR Method

The multiplex real‐time PCR reaction system was optimized using a primer and probe concentration gradient PCR test (Table S1). An annealing temperature of 62°C was confirmed through a temperature gradient PCR test (Table S2). The optimized multiplex real‐time PCR reaction was carried out in a final volume of 25.00 μL, containing 12.50 μL of qPCR Mix (Toyobo Co. Ltd., Osaka, Japan), 0.20 μM of each primer (Ovi‐F/R, Sus‐F/R and Gal‐F/R), 0.24 μM of Ana‐F/R primer, 0.10 μM of each probe (Ovi‐P, Sus‐P and Gal‐P), 0.12 μM of Ana‐P probe, 50 ng of DNA template, and molecular‐grade water. The thermal cycling conditions were as follows: an initial denaturation at 95°C for 10 min, followed by 40 cycles of 95°C for 10 s and 62°C for 30 s. Thus, a multiplex real‐time PCR method was developed for the detection of mutton, pork, chicken, and duck meat.

### 3.2 Specificity of the Multiplex Real‐Time PCR Method

**FIGURE 1:**
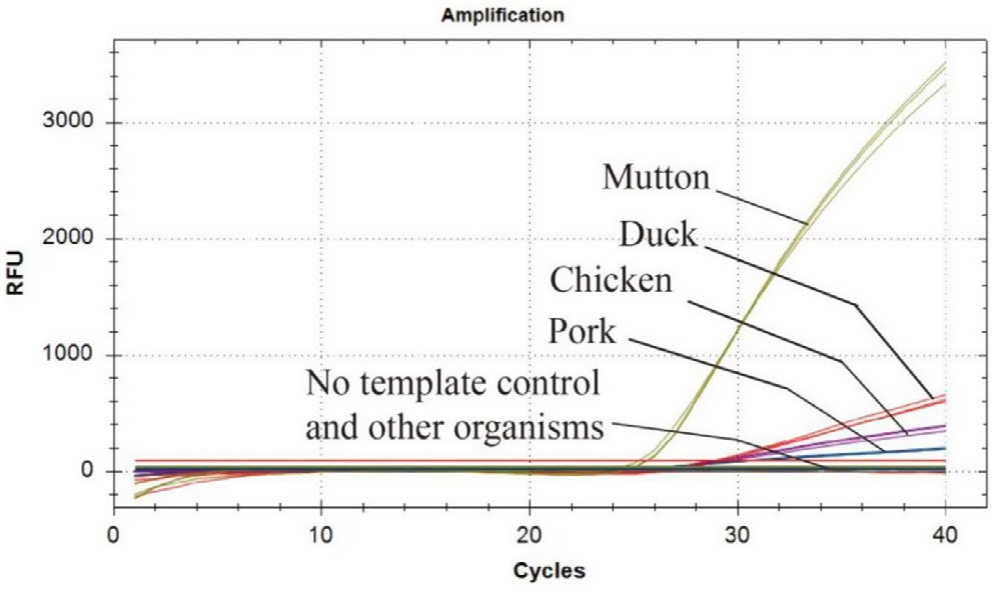
Specificity of developed multiplex real time PCR method.

The specificity of the primers and probes is critical for developing a multiplex real‐time PCR assay for species identification. DNA extracted from 23 samples, including animal species, plant mixtures, and microbial mixtures, was used to confirm the specificity of the primers and probes. Fluorescent signals were obtained only from mutton, pork, chicken, and duck, with no fluorescence signals observed for other non‐target organisms (Figure 1). These results demonstrated that the primers and probes exhibited high specificity.

### 3.3 Accuracy of the Multiplex Real‐Time PCR Method

Eleven reference samples were mixed to evaluate the accuracy of the established multiplex real‐time PCR method. DNA was extracted from these samples and used for the multiplex real‐time PCR assay. As shown in Table 2, the results of the multiplex real‐time PCR for the 11 reference samples were consistent with the predicted meat components, indicating that the established multiplex real‐time PCR method exhibited high accuracy.

**TABLE 2:** Detection results of the reference samples using the multiplex real‐time PCR method.

Samples	Meat content	Mean of Ct Value ± SD					Multiplex real‐time PCR results			
		Mutton	Pork	Chicken	Duck		Mutton	Pork	Chicken	Duck
A1	Mutton + Pork + Chicken + Duck	23.22 ± 0.02	21.44 ± 0.09	25.04 ± 0.03	25.06 ± 0.07		+	+	+	+
A2	Mutton + Pork + Chicken	22.88 ± 0.11	21.76 ± 0.09	24.65 ± 0.07	ND		+	+	+	−
A3	Mutton + Pork + Duck	23.73 ± 0.07	22.08 ± 0.18	ND	25.93 ± 0.21		+	+	−	+
A4	Mutton + Chicken + Duck	22.74 ± 0.28	ND	24.78 ± 0.14	25.82 ± 0.16		+	−	+	+
A5	Pork + Chicken + Duck	ND	20.97 ± 0.02	25.12 ± 0.8	24.28 ± 0.18		−	+	+	+
A6	Mutton + Pork	22.48 ± 0.06	22.97 ± 0.05	ND	ND		+	+	−	−
A7	Mutton + Chicken	22.50 ± 0.07	ND	24.17 ± 0.28	ND		+	−	+	−
A8	Mutton + Duck	22.47 ± 0.04	ND	ND	24.17 ± 0.17		+	−	−	+
A9	Pork + Chicken	ND	21.08 ± 0.12	24.64 ± 0.15	ND		−	+	+	−
A10	Pork + Duck	ND	21.47 ± 0.17	ND	25.00 ± 0.28		−	+	−	+
A11	Chicken + Duck	ND	ND	25.04 ± 0.18	25.15 ± 0.08		−	−	+	+

### 3.4 Sensitivity of the Multiplex Real‐Time PCR Method

Analytical sensitivity was expressed as the limit of detection (LOD) in the qualitative real‐time PCR. The LOD is defined as the lowest DNA concentration that produces a significant fluorescent signal (Kang 2019). In this study, the DNA concentration gradient used for sensitivity analysis included approximately 20, 10, 5, and 1 copies. Each concentration was tested in 60 replicates. According to the ENGL criteria (ENGL 2015), the LOD is the lowest concentration that yields at least 57 positive results out of 60 replicates. As shown in Table 3, the lowest DNA concentrations for mutton, pork, chicken, and duck were 10, 10, 10, and 20 copies, respectively, which met the LOD_95%_ principle.

**TABLE 3:** Amplification data used to determine the LOD of the established real‐time PCR method.

Samples	Target gene	Copies	DNA amount (ng)	Positive signals parallels/Total parallels	Mean of all Ct values	SD	RSD (%)
D1	*PHPAP*	20	0.058	60/60	32.25	0.27	0.84
		10	0.029	60/60	32.37	0.3	0.93
		5	0.014	56/60	35.04	2.41	6.88
		1	0.003	38/60	36.3	1.63	4.49
D2	*PRNP*	20	0.053	60/60	28.95	1.71	5.91
		10	0.027	60/60	32.14	1.62	5.04
		5	0.013	34/60	35.59	1.93	5.42
		1	0.003	25/60	35.73	2.49	6.97
D3	*TGF‐β3*	20	0.023	60/60	31.62	0.36	1.14
		10	0.011	60/60	33.05	0.55	1.66
		5	0.006	56/60	33.09	1.79	5.41
		1	0.001	30/60	34.41	1.39	4.04
D4	*ACTB*	20	0.026	60/60	34.58	1.91	5.52
		10	0.013	56/60	36.63	1.18	3.22
		5	0.006	54/60	37.06	1.43	3.86
		1	0.001	28/60	37.61	2.03	5.4

### 3.5 Application of the Multiplex Real‐Time PCR Method

The multiplex real‐time PCR method was used to detect meat components in 35 processed meat products, including mutton, pork, chicken, and duck. Label information shows the composition of the meat components. As shown in Table 4, all samples exhibited consistent label information and detection results, except for sample S23 (mutton rolls), which contained only pork. Additionally, the label information for sample S21 indicated that it contained pork and chicken, with pork content exceeding 90%. Since the multiplex real‐time PCR method is qualitative, it could not measure the exact pork content in S21.

**TABLE 4:** Applicability of the established multiplex real‐time PCR method in analyzing processed meat products.

Sample	Product type	Declaration	Mean of Ct Value ± SD				Multiplex real‐time PCR results			
			Mutton	Pork	Chicken	Duck	Mutton	Pork	Chicken	Duck
S2	Baconic	Pork	ND	22.18 ± 0.14	ND	ND	−	+	−	−
S3	Mutton	Mutton	20.75 ± 0.06	ND	ND	ND	+	−	−	−
S4	Chicken thigh	Chicken	ND	ND	23.12 ± 0.15	ND	−	−	+	−
S5	Luncheon meat	Pork	ND	22.33 ± 0.09	ND	ND	−	+	−	−
S7	Mutton	Mutton	20.75 ± 0.17	ND	ND	ND	+	−	−	−
S8	Chicken breast	Chicken	ND	ND	23.42 ± 0.15	ND	−	−	+	−
S9	Chicken sausage	Chicken	ND	ND	24.14 ± 0.04	ND	−	−	+	−
S10	Luncheon meat	Pork+ Chicken	ND	23.92 ± 0.16	27.65 ± 0.12	ND	−	+	+	−
S11	Luncheon meat	Pork	ND	25.48 ± 0.11	ND	ND	−	+	−	−
S13	Chicken sausage	Chicken	ND	ND	24.46 ± 0.06	ND	−	−	+	−
S15	Chicken breast	Chicken	ND	ND	24.28 ± 0.01	ND	−	−	+	−
S17	Mutton rolls	Mutton	21.59 ± 0.12	ND	ND	ND	+	−	−	−
S18	Hot dog meat	Chicken	ND	ND	23.34 ± 0.09	ND	−	−	+	−
S19	Ham	Pork+ Chicken	ND	24.51 ± 0.17	27.78 ± 0.21	ND	−	+	+	−
S20	Ham	Pork+ Chicken	ND	26.62 ± 0.17	27.83 ± 0.13	ND	−	+	+	−
S21	Luncheon meat	Pork+ Chicken (Pork > 90%)	ND	25.34 ± 0.05	28.60 ± 0.05	ND	−	+	+	−
S23	Mutton rolls	Mutton + Pork	ND	23.05 ± 0.18	ND	ND	−	+	−	−
S29	Mutton rolls	Mutton	21.22 ± 0.10	ND	ND	ND	+	−	−	−
S31	Shish kebab	Mutton	23.10 ± 0.05	ND	ND	ND	+	−	−	−
S32	Shish kebab	Mutton	25.00 ± 0.10	ND	ND	ND	+	−	−	−
S33	Shish kebab	Mutton	23.24 ± 0.17	ND	ND	ND	+	−	−	−
S34	Mutton rolls	Mutton	21.97 ± 0.26	ND	ND	ND	+	−	−	−
S35	Mutton rolls	Mutton	21.93 ± 0.12	ND	ND	ND	+	−	−	−
S36	Mutton rolls	Mutton	23.20 ± 0.12	ND	ND	ND	+	−	−	−
S37	Mutton rolls	Mutton+ Pork	25.55 ± 0.16	22.78 ± 0.00	ND	ND	+	+	−	−
S38	Mutton rolls	Mutton	22.46 ± 0.05	ND	ND	ND	+	−	−	−
S39	Mutton rolls	Mutton + Pork	30.12 ± 0.28	24.19 ± 0.11	ND	ND	+	+	−	−
S40	Mutton rolls	Mutton	21.68 ± 0.06	ND	ND	ND	+	−	−	−
S41	Mutton rolls	Mutton	21.24 ± 0.02	ND	ND	ND	+	−	−	−
S42	Mutton rolls	Mutton	21.22 ± 0.13	ND	ND	ND	+	−	−	−
S49	Mutton rolls	Mutton	21.45 ± 0.10	ND	ND	ND	+	−	−	−
S50	Mutton rolls	Mutton	21.42 ± 0.07	ND	ND	ND	+	−	−	−
S51	Duck neck	Duck	ND	ND	ND	24.39 ± 0.08	−	−	−	+
S52	Duck wing	Duck	ND	ND	ND	23.64 ± 0.08	−	−	−	+
S53	Duck leg	Duck	ND	ND	ND	24.88 ± 0.20	−	−	−	+

## 4 Discussion

With the development of meat‐processing technology, traditional sensory methods are no longer sufficient to distinguish between different meat components. Modern molecular biology techniques are widely used to identify meat components. To date, real‐time PCR has remained the main technology for meat detection (Hossain et al. 2023). Numerous studies have reported real‐time PCR methods for detecting constituents in processed meat products (Kim et al. 2023; Konduru et al. 2021; Köppel et al. 2020; Zhu et al. 2021). Given the prevalence of mutton adulteration with pork, chicken, or duck in China (Yang et al. 2022), a multiplex real‐time PCR method for detecting mutton, pork, chicken, and duck can efficiently screen for undeclared meat components in mutton products. Therefore, this method has significant potential for the detection of adulteration in processed mutton products.

Currently, target genes for real‐time PCR methods primarily include mitochondrial and nuclear genes. Mitochondrial genes such as *12S rRNA* (Li et al. 2019), *16S rRNA* (Li et al. 2019), *cytochrome b* (Ali et al. 2015; Hossain et al. 2017; Kim and Kim 2019), *NADH dehydrogenase* (Ali et al. 2015; Hossain et al. 2017), *cytochrome c oxidase* (Li et al. 2019), and *ATPase subunit coding genes* (Ali et al. 2015) are commonly used to detect meat adulteration. However, the nuclear genome contains a large amount of information with a complex structure, complicating the screening of target genes. Consequently, fewer reports focus on detection methods based on nuclear genes (Floren et al. 2015; Iwobi et al. 2015; Li et al. 2021; Nixon et al. 2015; Wang et al. 2019, 2021). In this study, to establish a stable multiplex real‐time PCR method, the whole‐genome sequence of each animal was analyzed to identify single‐copy nuclear genes. Finally, the nuclear genes *PHPAP*, *PRNP*, *TGF‐β3*, and *ACTB* were selected as target genes for developing the multiplex real‐time PCR methods.

Method validation is a critical step in producing reliable analytical data. In this study, we utilized stable and reliable CRMs to validate the specificity, accuracy, and LOD. A CRM is accompanied by a certificate that verifies it has been characterized using a metrologically valid procedure for one or more specified properties, which are carefully specified and documented (Mattsson and Zetterberg 2012). Using CRMs to validate the method improved the reliability of the tests with respect to its specificity, accuracy, and sensitivity. Unlike previous studies, we employed a diverse range of experimental materials to assess specificity and minimize the risk of cross‐reactivity with non‐target species. Additionally, reference samples prepared with varying CRM weights were used to validate the accuracy, which is essential for reliable application of the method. Finally, the LOD was determined according to the ENGL criteria (ENGL 2015), ensuring a rigorous and scientifically sound approach to confirm the minimum detectable DNA concentration. According to ENGL criteria (ENGL 2015), samples with CT values below the LOD are considered “positive,” while values equal to or above the LOD are “negative.” Based on this criterion, 34 out of 35 tested processed meat samples showed consistent labeling and detection results. However, our review of product labels revealed that most declarations were qualitative, allowing the use of substantial proportions of low‐cost meat ingredients without precise disclosure—highlighting the need for complementary quantitative methods in future work. For instance, mutton rolls S37 and S39 declared the presence of both mutton and pork; however, these products likely contained only a small amount of mutton, with pork comprising the majority of their composition. To effectively prevent meat adulteration and promote healthy development of the meat industry, we recommend mandatory quantitative labeling of meat components.

The applicability of the multiplex real‐time PCR method for species identification is essential for its practical deployment in the identification of meat adulteration. Processed meat products often contain complex matrices—such as seasonings, fats, and additives—which may inhibit DNA extraction or PCR amplification, potentially affecting detection accuracy. In this study, products like lunch meats, hot dogs, hams, and lamb kebabs (Table 4) exhibited such complex compositions, as evidenced by their ingredient lists. Despite these challenges, the method established here successfully identified the meat species without apparent matrix interference. However, the method also has several limitations that warrant discussion. First, while qualitative real‐time PCR allows for sensitive meat species detection, it does not provide quantitative information about the proportion of each meat species present. This limits the ability to assess the extent of adulteration or compliance with labeling laws that require quantitative declarations. Second, the difficulty in determining results when Ct values are close to the LOD—such as S21 (28.60) and S39 (30.12). It is recommended that the proportion of high CT species be determined by quantitative methods to identify adulteration. Third, although no matrix interference was observed in this study, the robustness of the method against diverse matrices was not systematically evaluated across different product types or processing conditions. Therefore, its applicability to products with highly degraded DNA, excessive fat content, or strong PCR inhibitors remains to be further validated.

Although the current method is designed for qualitative detection, the development of a reliable quantitative assay is essential to meet the increasing demands for precise measurement in meat product authentication. In future work, we plan to develop a singleplex real‐time PCR assay with improved specificity and sensitivity for quantitative detection of different meat species. By minimizing fluorescence interference and optimizing amplification conditions, this quantitative system will complement the current qualitative screening, forming a two‐tier detection strategy that enhances both accuracy and applicability in meat safety monitoring.

## Author Contributions

**Lijuan Chang:** conceptualization (equal), data curation (equal), methodology (equal), writing – original draft (equal). **Chengping Fu:** investigation (equal). **Mengxue Deng:** project administration (equal). **Yuanhong Li:** supervision (equal), writing – review and editing (equal).

## Conflicts of Interest

The authors declare no conflicts of interest.

## Supporting information


Data S1.

## Data Availability

Data will be made available on request.
