# Supplementary material for: A Multiplex Real‐Time PCR for Qualitative Detection of Mutton, Pork, Chicken, and Duck in Processed Meat Products
Source: Food Sci Nutr. 2025 Jul 14;13(7):e70590. doi: 10.1002/fsn3.70590 (PMC12257148; doi:10.1002/fsn3.70590)
Supplement: Supplementary file 1 — Data S1. [file FSN3-13-e70590-s001.docx]

Suppl. Table 1 Amplification data used to optimise primer and probe concentrations for multiplex real-time PCR.

| **Gradient** | | **Beef** | | **pork** | | **Chicken** | | **Duck** |
| --- | --- | --- | --- | --- | --- | --- | --- | --- |
| CG1  CG2  CG3  CG4 | NA  30.81±0.07  30.47±0.06  30.29±0.18 | | 31.17±0.24  29.66±0.44  28.97±0.18  28.86±0.64 | | 30.38±0.05  29.04±0.19  28.44±0.22  28.32±0.41 | | 32.40±0.17  31.55±0.23  31.27±0.23  30.44±0.53 | |

Suppl. Table 2 Amplification data used to optimise temperature for multiplex real-time PCR.

| **Gradient** | | **Beef** | | **pork** | | **Chicken** | | **Duck** |
| --- | --- | --- | --- | --- | --- | --- | --- | --- |
| TG1  TG2  TG3  TG4 | 31.41±0.08  29.94±0.09  29.61±0.16  29.37±0.15 | | 28.92±0.27  28.98±0.21  28.43±0.20  28.01±0.23 | | 28.91±0.27  28.86±0.17  28.55±0.17  28.10±0.25 | | 32.11±0.56  31.52±0.17  31.55±0.53  30.84±0.29 | |
